# 芦可替尼联合糖皮质激素一线治疗急性移植物抗宿主病对巨细胞病毒激活的影响

**DOI:** 10.3760/cma.j.issn.0253-2727.2022.09.004

**Published:** 2022-09

**Authors:** 双义 邢, 坤 钱, 英欣 赵, 博 彭, 晶晶 杨, 立萍 窦, 代红 刘

**Affiliations:** 解放军医学院、解放军总医院、解放军总医院第五医学中心血液病医学部，北京 100853 Medical School of Chinese PLA, Chinese PLA General Hospital, Department of Hematology in the Fifth Medical Center of PLA General Hospital, Beijing 100853, China

**Keywords:** 异基因造血干细胞移植, 急性移植物抗宿主病, 芦可替尼, 巨细胞病毒, Allogeneic hematopoietic stem cell transplantation, Acute graft-versus-host disease, Ruxolitinib, Cytomegalovirus

## Abstract

**目的:**

观察芦可替尼联合糖皮质激素一线治疗急性移植物抗宿主病（GVHD）对巨细胞病毒（CMV）激活的影响。

**方法:**

回顾性分析2018年8月至2020年9月期间195例在解放军总医院第一医学中心血液病科进行异基因造血干细胞移植患者的临床资料，根据急性GVHD发生情况分为无、Ⅰ度、Ⅱ～Ⅳ度、Ⅲ/Ⅳ度急性GVHD组，根据急性GVHD一线治疗方案分为联合方案组（芦可替尼联合糖皮质激素）及经典方案组（单用糖皮质激素）。分析移植后90 d及180 d各组CMV激活累积发生率、CMV激活时长及转阴时长，比较联合方案组及经典方案组总生存率及无病生存率。

**结果:**

195例患者中无急性GVHD 64例（32.8％）、Ⅰ度急性GVHD 30例（15.4％）、Ⅱ～Ⅳ度急性GVHD 101例（51.8％）、Ⅲ/Ⅳ度急性GVHD 14例（7.2％）。联合方案组、经典方案组Ⅰ～Ⅳ度急性GVHD患者在移植后90 d 及180 d CMV激活累积发生率差异无统计学意义（*P*值均>0.05），且CMV激活时长及转阴时长差异也无统计学意义（*P*值均>0.05）。进一步对两种方案一线治疗Ⅱ～Ⅳ度及Ⅲ/Ⅳ度急性GVHD进行分析，发现在移植后90 d 及180 d CMV激活累积发生率、激活时长及转阴时长差异均无统计学意义（*P*值均>0.05）。此外，两组急性GVHD患者移植后2年总生存率及无病生存率差异均无统计学意义（*P*值均>0.05）。

**结论:**

芦可替尼联合糖皮质激素一线治疗急性GVHD不增加CMV激活的风险。

## Abstract (en)

**Objective:**

To observe the effect of ruxolitinib combined with glucocorticoid on cytomegalovirus (CMV) activation in patients with acute graft-versus-host disease (aGVHD).

**Methods:**

The clinical data of 195 patients who underwent allogeneic hematopoietic stem cell transplantation in the Department of Hematology of the First Medical Center of the People's Liberation Army General Hospital from August 2018 to September 2020 were retrospectively analyzed. According to the severity of aGVHD, the patients were divided into the non-GVHD group, aGVHD grade Ⅰ group, aGVHD grade Ⅱ-Ⅳ group, and aGVHD grade Ⅲ/Ⅳ group. In addition, they were classified into two subgroups according to the first-line treatment regimen for aGVHD: combined regimen group (ruxolitinib combined with glucocorticoid) and classical regimen group (glucocorticoid alone). The cumulative incidence of CMV activation, the duration of CMV activation, and the duration of CMV negativity in each subgroup at 90 and 180 days after transplantation were analyzed. The overall survival and disease-free survival rates of patients in both regimens were compared.

**Results:**

Sixty-four (32.8％) patients in the group did not develop aGVHD. The numbers of patients with grade Ⅰ, Ⅱ-Ⅳ, and Ⅲ/Ⅳ aGVHD were 30 (15.4％), 101 (51.8％), and 14 (7.2％), respectively. Compared with patients in the classical regimen, no significant difference was observed in the cumulative incidence of CMV activation, duration of CMV activation, and duration of CMV negativity in patients with grade Ⅰ-Ⅳ aGVHD in the combined regimen at 90 and 180 days after transplantation (*P*>0.05). Further analysis of patients with grade Ⅱ-Ⅳ and Ⅲ/Ⅳ aGVHD showed that the cumulative incidence of CMV activation, duration of CMV activation, and duration of CMV negativity did not show significant difference between the two treatment regimens (*P*>0.05). In addition, there was no significant difference in the overall survival and disease-free survival rates of patients in both regimens (*P*>0.05).

**Conclusion:**

Ruxolitinib combined with glucocorticoid as the first-line therapy for aGVHD did not increase the risk of CMV activation.

异基因造血干细胞移植（allo-HSCT）是治疗恶性血液病及重型再生障碍性贫血的重要方法，但常伴发移植物抗宿主病（GVHD）和巨细胞病毒（CMV）激活等多种合并症。国内文献报道，在单倍体造血干细胞移植中Ⅱ～Ⅳ度、Ⅲ/Ⅳ度急性GVHD发生率分别为18.5％～43.9％、7.9％～13.8％[1]。急性GVHD是移植后非复发死亡的主要原因[2]，目前指南推荐的一线治疗是甲泼尼龙1～2 mg·kg^−1^·d^−1^[1]，但其疗效有限[3]。芦可替尼是一种选择性JAK 1/2抑制剂，2017年美国FDA批准芦可替尼用于糖皮质激素耐药急性GVHD的二线治疗[4]–[5]。本中心首次将芦可替尼联合糖皮质激素用于急性GVHD一线治疗，也取得了良好疗效[6]。

allo-HSCT后1年CMV激活的累积发生率为55％～67％[7]–[9]，CMV病发生率为5％～10％[10]，CMV激活是移植后患者非复发死亡的主要原因[10]–[11]。芦可替尼用于糖皮质激素耐药急性GVHD二线治疗的数据提示其可能对CMV激活有促进作用[5]。目前芦可替尼联合糖皮质激素用于急性GVHD一线治疗对CMV激活的影响尚无报道。本研究纳入2018年8月至2020年9月在解放军总医院第一医学中心行allo-HSCT的195例患者，探讨芦可替尼联合糖皮质激素方案一线治疗急性GVHD对CMV激活的影响。

## 病例与方法

一、病例

本研究连续性纳入2018年8月至2020年9月在解放军总医院第一医学中心血液病科进行allo-HSCT的195例患者，采集人口学信息、疾病信息、移植信息、移植合并症（急性GVHD与CMV激活）及转归等数据进行回顾性分析。纳入标准：①具有allo-HSCT适应证的恶性血液病和重型再生障碍性贫血患者；②无allo-HSCT禁忌证；③患者本人及家属签署移植知情同意书。

二、急性GVHD的预防、诊断及治疗

应用环孢素A（CsA）、甲氨蝶呤（MTX）、霉酚酸酯（MMF）预防急性GVHD[12]。单倍体移植：CsA 2 mg/kg 每日2次，移植前10 d至移植后3个月（维持血药谷浓度180～250 µg/L），在未出现GVHD的情况下于移植后6个月逐渐减量至停药；霉酚酸酯500 mg每日2次，移植前10 d～移植后45 d；移植后1 d，甲氨蝶呤15 mg·m^−2^·d^−1^静脉滴注，移植后3、6、11 d，甲氨蝶呤10 mg·m^−2^·d^−1^静脉滴注。同胞全相合移植：CsA 2 mg/kg每日2次，移植前10 d至移植后2个月（维持血药谷浓度180～250 µg/L），在未发生GVHD的情况下于移植后6个月逐渐减量至停药；霉酚酸酯500 mg每日2次，移植前10 d～移植后30 d；在移植后1 d，甲氨蝶呤15 mg·m^−2^·d^−1^静脉滴注，移植后3、6、11 d，甲氨蝶呤10 mg·m^−2^·d^−1^静脉滴注。

主要依赖皮肤、胃肠道、肝脏受累所表现的症状诊断急性GVHD。根据改良Glucksberg标准[13]–[14]分为Ⅰ～Ⅳ度。在纳入研究的195例患者中，73例急性GVHD患者一线治疗采用目前指南推荐的经典方案（甲泼尼龙1～2 mg·kg^−1^·d^−1^）。根据我中心一项前瞻性临床试验（ClinicalTrials: NCT04397367）安排，58例急性GVHD患者一线治疗方案为芦可替尼联合糖皮质激素方案：①此类患者均为明尼苏达急性GVHD预后评分[15]为高危或评分为标危的同时生物标志物检测[16]–[18]为中危、高危（评分分别为2、3分）；②应用联合方案的方法：使用甲泼尼龙1 mg·kg^−1^·d^−1^，同时给予芦可替尼5 mg/d，随着急性GVHD消退，芦可替尼在2个月内逐渐减量至停用。若一线治疗无效，给予巴利昔单抗、甲氨蝶呤、霉酚酸类药物等二线治疗药物。

三、CMV激活及转阴的定义

CMV激活定义：在移植后患者血液中检测到CMV阳性（CMV-DNA载量>1000拷贝/ml）[7]。

激活时长定义：CMV阳性时间的总和。CMV转阴定义：CMV转阴后持续14 d以上。转阴时长定义：持续14 d以上的CMV阴性时间。

四、CMV激活预防、监测及治疗

所有患者接受更昔洛韦（移植前10 d至移植前3 d）、阿昔洛韦或伐昔洛韦（移植后1 d至1.5年）作为病毒预防方案。采用荧光免疫定量聚合酶链反应（PCR）检测血液中CMV-DNA定量来监测CMV激活，移植后90 d内每周至少检测1次，90 d～180 d期间每2周检测1次。检测到CMV-DNA>1.0×10^4^拷贝/ml或连续2次检测到>1.0×10^3^拷贝/ml时，给予更昔洛韦5 mg/kg每12 h 1次静脉滴注，若治疗无效或血象偏低则予以膦甲酸钠60 mg/kg每8 h 1次静脉滴注，至CMV-DNA连续2次≤1.0×10^3^拷贝/ml后，改以阿昔洛韦或伐昔洛韦预防。

五、随访

通过电话随访、查阅住院/门诊病历获取患者随访资料。末次随访日期为2021年12月10日。中位随访时间为710（369～1120）d。

六、统计学处理

通过R 4.0.5软件和cmprsk软件包进行竞争性风险分析（无CMV感染所致死亡、失访被认为是CMV激活的竞争事件），计算各亚组CMV不同类型激活情况的累积发生率。使用SPSS 26.0软件进行统计分析，计数资料比较采用*χ*^2^检验或精确概率法，非正态分布的计量资料采用秩和检验对各组进行比较，总生存（OS）及无病生存（DFS）均使用Kaplan-Meier方法进行分析。以*P*<0.05为差异有统计学意义。

## 结果

一、一般资料

共195例患者纳入研究，男146例，女49例。年龄小于40岁患者共124例（63.6％），中位年龄33（9～63）岁；无急性GVHD组64例（32.8％）、Ⅰ度急性GVHD组30例（15.4％）、Ⅱ～Ⅳ度急性GVHD组101例（51.8％）、Ⅲ/Ⅳ度急性GVHD组14例（7.2％）。基本特征详见表1。在移植后90 d、180 d 的CMV激活累积发生率分别为69.7％、71.3％。在195例患者中23例出现CMV病，占11.8％。

**表1:** 195例异基因造血干细胞移植患者的基本特征

临床特征	结果
患者年龄［岁，*M*（范围）］	33（9～63）
患者年龄分组［例（％）］	
<40岁	124（63.6）
≥40岁	71（36.4）
急性GVHD发生情况［例（％）］	
无	64（32.8）
Ⅰ度	30（15.4）
Ⅱ～Ⅳ度	101（51.8）
患者性别［例（％）］	
男性	146（74.9）
女性	49（25.1）
疾病类型［例（％）］	
AML	102（52.3）
MDS	15（7.7）
CML	3（1.5）
ALL/LBL	57（29.2）
NHL	6（3.1）
MPAL	3（1.5）
AA	5（2.6）
MDS/MPN	4（2.1）
移植前疾病状态［例（％）］	
完全缓解	135（69.2）
非完全缓解	47（24.1）
未治疗	8（4.1）
非恶性肿瘤	5（2.6）
预处理方案含ATG［例（％）］	
否	1（0.5）
是	194（99.5）
预处理方案［例（％）］	
不含氟达拉滨	186（95.4）
BU/CY	165（84.6）
TBI/CY	21（10.8）
含氟达拉滨	9（4.6）
供者类型［例（％）］	
同胞全相合供者	41（21.0）
非血缘供者	11（5.6）
单倍体供者	143（73.3）
供者年龄［岁，*M*（范围）］	36（8~59）
供者年龄分组［例（％）］	
<40岁	113（57.9）
≥40岁	82（42.1）
供患者ABO血型组合［例（％）］	
相合	105（53.8）
主要不合	42（21.5）
次要不合	32（16.4）
主次均不合	16（8.2）
供患者性别组合［例（％）］	
女供女	19（9.7）
女供男	49（25.1）
男供女	30（15.4）
男供男	97（49.8）
移植前患者CMV血清抗体类型［例（％）］	
lgM^−^IgG^＋^	192（98.5）
lgM^−^IgG^−^	2（1.0）
lgM^＋^IgG^＋^	1（0.5）
移植前供者CMV血清抗体类型［例（％）］	
lgM^−^IgG^＋^	172（88.2）
lgM^−^IgG^−^	6（3.1）
lgM^＋^IgG^＋^	1（0.5）
未知	16（8.2）
移植物单个核细胞［×10^8^/kg，*M*（范围）］	10.71（3.68～30.45）
移植物CD34^+^细胞［×10^6^/kg，*M*（范围）］	4.38（1.76～22.41）

注：AML：急性髓系白血病；MDS：骨髓增生异常综合征；CML：慢性髓性白血病；ALL/LBL：急性淋巴细胞白血病/淋巴母细胞淋巴瘤；NHL：非霍奇金淋巴瘤；MPAL：急性混合白血病；AA：再生障碍性贫血；MDS：骨髓增生异常综合征；MPN：骨髓增殖性肿瘤；ATG：抗人T淋巴细胞兔免疫球蛋白；BU：白消安；CY：环磷酰胺；TBI：全身照射治疗

二、两种急性GVHD一线治疗方案对移植后CMV激活的影响

经典方案、联合方案组移植后90 d CMV激活累积发生率分别为76.7％、82.8％（*P*＝0.625），移植后180 d CMV激活累积发生率分别为78.1％、84.5％（*P*＝0.594）。在Ⅱ～Ⅳ度急性GVHD患者中，经典方案组、联合方案组移植后90 d CMV激活累积发生率分别为80.4％、82.2％（*P*＝0.864），移植后180 d CMV激活累积发生率分别为80.4％、84.4％（*P*＝0.968）。此外，在Ⅲ～Ⅳ度急性GVHD患者中，经典方案组、联合方案组移植后90 d、180 d CMV激活累积发生率分别为66.7％、75.0％（*P*＝0.843）。在Ⅰ～Ⅳ度急性GVHD患者中，两组移植后90 d、180 d 的CMV激活时长、转阴时长差异均无统计学意义（*P*值均>0.05）；在Ⅱ～Ⅳ度急性GVHD患者中，两组移植后180 d的CMV激活时长及转阴时长差异均无统计学意义（*P*值均>0.05）。详见表2。

**表2:** 两种急性GVHD一线治疗方案对异基因造血干细胞移植后巨细胞病毒（CMV）激活的影响

组别	CMV激活累积发生率		CMV激活时长		CMV转阴时长	
	率［%（95%*CI*）］	*P*值	值［d，*M*（范围）］	*P*值	值［d，*M*（范围）］	*P*值
Ⅰ～Ⅳ度急性GVHD						
+90 d		0.625		0.470		0.372
经典方案	76.7（65.0～84.9）		25.0（3.0～63.0）		42.0（18.0～49.0）	
联合方案	82.8（69.9～90.5）		25.0（3.0～58.0）		23.0（18.0～35.0）	
+180 d		0.594		0.343		0.157
经典方案	78.1（66.5～86.1）		29.5（3.0～129.0）		41.0（15.0～77.0）	
联合方案	84.5（71.8～91.8）		25.0（3.0～144.0）		27.5（18.0～71.0）	
Ⅱ～Ⅳ度急性GVHD						
+90 d		0.864		0.551		
经典方案	80.4（66.9～88.8）		25.0（3.0～60.0）		−	−
联合方案	82.2（66.9～90.9）		24.5（3.0～58.0）		−	
+180 d		0.968		0.498		0.428
经典方案	80.4（66.9～88.8）		29.5（3.0～84.0）		43.0（15.0～77.0）	
联合方案	84.4（69.3～92.5）		25.0（3.0～131.0）		28.0（18.0～71.0）	
Ⅲ/Ⅳ度急性GVHD						
+90 d		0.843		0.439		
经典方案	66.7（12.2～92.5）		7.0（4.0～20.0）		−	−
联合方案	75.0（23.3～94.5）		23.0（3.0～41.0）		−	
+180 d		0.843		0.606		
经典方案	66.7（12.2～92.5）		11.0（7.0～20.0）		−	−
联合方案	75.0（23.3～94.5）		23.0（3.0～41.0）		−	

注：GVHD：移植物抗宿主病；联合方案：芦可替尼联合糖皮质激素；经典方案：单用糖皮质激素；−：不适用

三、两种急性GVHD一线治疗方案移植预后比较

至随访截至，在Ⅰ～Ⅳ度、Ⅱ～Ⅳ度及Ⅲ～Ⅳ度急性GVHD患者中，两种方案组的2年OS率及DFS率差异无统计学意义（*P*值均>0.05）。在Ⅰ～Ⅳ度急性GVHD患者中，联合方案、经典方案组的2年OS率分别为（64.8±12.5）％、（60.7±11.8）％（*χ*^2^＝0.063，*P*＝0.803），2年DFS率分别为（63.1± 12.5）％、（58.5±12.5）％（*χ*^2^＝0.029，*P*＝0.866）；在Ⅱ～Ⅳ度及Ⅲ/Ⅳ度急性GVHD患者中，联合方案与经典方案亚组2年OS及DFS差异均无统计学意义（*P*值均>0.05）。详见表3。

**表3:** 两种方案一线治疗急性GVHD患者的异基因造血干细胞移植后2年生存率比较

组别	例数	2年OS率		2年DFS率	
		值［%（95%*CI*）］	*P*值	值［%（95%*CI*）］	*P*值
Ⅰ～Ⅳ度急性GVHD			0.803		0.866
经典方案	73	60.7（48.9～72.5）		58.5（46.0～71.0）	
联合方案	58	64.8（52.3～77.3）		63.1（50.6～75.6）	
Ⅱ～Ⅳ度急性GVHD			0.763		0.684
经典方案	56	59.4（45.7～73.1）		56.2（41.1～71.3）	
联合方案	45	58.7（44.0～73.4）		56.5（41.6～71.4）	
Ⅲ/Ⅳ度急性GVHD			0.357		0.640
经典方案	6	50.0（12.0～88.0）		50.0（12.0～88.0）	
联合方案	8	75.0（35.0～97.0）		62.5（24.0～93.0）	

注：GVHD：移植物抗宿主病；联合方案：芦可替尼联合糖皮质激素；经典方案：单用糖皮质激素；OS：总生存；DFS：无病生存

## 讨论

芦可替尼的不良反应包括血液学毒性和感染[19]。有报道显示，在糖皮质激素耐药的急性GVHD的二线治疗中，芦可替尼组CMV感染发生率高于对照组（26％对21％）[5]。本研究通过对195例allo-HSCT患者临床资料的分析探讨芦可替尼联合糖皮质激素一线治疗对急性GVHD患者CMV激活的影响，与单用糖皮质激素相比，芦可替尼联合糖皮质激素方案并不会显著增加CMV激活累积发生率、不会延长CMV激活时长及缩短转阴时长，且不会显著影响患者的移植预后（2年OS及DFS），提示芦可替尼联合糖皮质激素一线治疗急性GVHD不增加CMV激活的风险。

国内外研究报道显示，allo-HSCT移植后患者1年CMV激活累积发生率可达55.0％～67.0％[7]–[9]，本组195例患者移植后90 d、180 d的CMV激活累积发生率分别为69.7％、71.3％。本研究中，在移植后180 d内出现CMV激活患者中，有97.9％的患者CMV首次激活发生于移植后90 d内。Sahin等[20]亦报道移植后患者CMV激活多发生在移植后3个月内，提示移植后罹患CMV激活的主要时期是免疫重建尚不完善的移植后前3个月。大多数移植中心在移植后早期以每周1～2次监测CMV-DNA，并在一定界值之上采取抢先治疗策略来降低CMV病发病率，但是由于CMV病患者的死亡率仍然较高，故而CMV病是最被临床关注的CMV感染类型。国内外文献报道CMV病发生率为5％～15.7％[10],[21]，本组病例CMV病发生率为11.8％。由于病例数有限，我们没有进一步分析两种方案下CMV病发生率是否存在统计学差异，未来可通过更大样本量来进一步探讨。

与以往多侧重于探讨CMV激活的危险因素或急性GVHD与CMV激活发生率关系的研究[22]–[23]不同，本研究从多个时间点及多个角度首次探讨了急性GVHD不同治疗方案对CMV激活的影响。首先，在本研究中我们分别从两个时间点（移植后90 d及180 d）去探讨两种方案对CMV激活的影响。其次，本研究在定义CMV激活及CMV转阴后，从CMV激活累积发生率、激活时长及转阴时长多个角度来比较两种方案对CMV激活的影响，从而更有力地证明，相较于指南推荐的经典方案（甲泼尼龙1～2 mg·kg^−1^·d^−1^），联合方案（芦可替尼联合糖皮质激素）一线治疗急性GVHD不增加CMV激活的风险。

综上所述，本组病例结果显示，相较于单用糖皮质激素的经典方案，芦可替尼联合糖皮质激素方案对移植后90 d、180 d的CMV激活累积发生率、CMV激活时长及转阴时长无明显影响；两种方案下各程度急性GVHD患者的2年OS及DFS差异无统计学意义，且联合方案一线治疗Ⅲ/Ⅳ度急性GVHD患者似乎有更大的生存获益。上述结论尚待多中心前瞻随机对照研究结果加以验证。
